# Association between pretreatment emotional distress and immune checkpoint inhibitor response in non-small-cell lung cancer

**DOI:** 10.1038/s41591-024-02929-4

**Published:** 2024-05-13

**Authors:** Yue Zeng, Chun-Hong Hu, Yi-Zheng Li, Jian-Song Zhou, Shu-Xing Wang, Meng-Dong Liu, Zhen-Hua Qiu, Chao Deng, Fang Ma, Chun-Fang Xia, Fei Liang, Yu-Rong Peng, Ao-Xi Liang, Sheng-Hao Shi, Shi-Jiao Yao, Jun-Qi Liu, Wen-Jie Xiao, Xiao-Qiao Lin, Xin-Yu Tian, Ying-Zhe Zhang, Zhuo-Ying Tian, Ji-An Zou, Yun-Shu Li, Chao-Yue Xiao, Tian Xu, Xiao-Jie Zhang, Xiao-Ping Wang, Xian-Ling Liu, Fang Wu

**Affiliations:** 1 Department of Oncology, The Second Xiangya Hospital, Central South University, Changsha, China; 2 National Clinical Research Center for Mental Disorders, and National Center for Mental Disorders, The Second Xiangya Hospital of Central South University, Changsha, China; 3 Hunan Cancer Mega-Data Intelligent Application and Engineering Research Centre, Changsha, China; 4 Key Laboratory of Molecular Radiation Oncology Hunan Province, Xiangya Hospital, Central South University, Changsha, China; 5 Xiangya School of Medicine, Central South University, Changsha, China; 6 Department of Psychology, University of Washington, Seattle, WA USA; 7 Department of Biostatistics, Zhongshan Hospital, Fudan University, Shanghai, China; 8 Hunan Key Laboratory of Tumor Models and Individualized Medicine, The Second Xiangya Hospital, Central South University, Changsha, China; 9 Hunan Key Laboratory of Early Diagnosis and Precision Therapy in Lung Cancer, The Second Xiangya Hospital, Central South University, Changsha, China; 10 FuRong Laboratory, Changsha, China

**Keywords:** Non-small-cell lung cancer, Immunotherapy, Emotion, Risk factors, Prognostic markers

## Abstract

Emotional distress (ED), commonly characterized by symptoms of depression and/or anxiety, is prevalent in patients with cancer. Preclinical studies suggest that ED can impair antitumor immune responses, but few clinical studies have explored its relationship with response to immune checkpoint inhibitors (ICIs). Here we report results from cohort 1 of the prospective observational STRESS-LUNG study, which investigated the association between ED and clinical efficacy of first-line treatment of ICIs in patients with advanced non-small-cell lung cancer. ED was assessed by Patient Health Questionnaire-9 and Generalized Anxiety Disorder 7-item scale. The study included 227 patients with 111 (48.9%) exhibiting ED who presented depression (Patient Health Questionnaire-9 score ≥5) and/or anxiety (Generalized Anxiety Disorder 7-item score ≥5) symptoms at baseline. On the primary endpoint analysis, patients with baseline ED exhibited a significantly shorter median progression-free survival compared with those without ED (7.9 months versus 15.5 months, hazard ratio 1.73, 95% confidence interval 1.23 to 2.43, *P* = 0.002). On the secondary endpoint analysis, ED was associated with lower objective response rate (46.8% versus 62.1%, odds ratio 0.54, *P* = 0.022), reduced 2-year overall survival rate of 46.5% versus 64.9% (hazard ratio for death 1.82, 95% confidence interval 1.12 to 2.97, *P* = 0.016) and detriments in quality of life. The exploratory analysis indicated that the ED group showed elevated blood cortisol levels, which was associated with adverse survival outcomes. This study suggests that there is an association between ED and worse clinical outcomes in patients with advanced non-small-cell lung cancer treated with ICIs, highlighting the potential significance of addressing ED in cancer management. ClinicalTrials.gov registration: NCT05477979.

## Abstract (web summary)

In a prospective observational study, symptoms of anxiety and/or depression were associated with worse response to first-line treatment with immunotherapy in patients with advanced non-small-cell lung cancer.

## Main

Immune checkpoint inhibitors (ICIs) have ushered in a remarkable advancement in the management of advanced non-small-cell lung cancer (NSCLC)^1^. Nevertheless, only 15–30% of patients will have long-lasting benefits^2^. Identifying resistance mechanisms of ICIs and predicting treatment efficacy are imperative^3^. While some predictive biomarkers have been identified, such as tumor programmed cell death ligand-1 (PD-L1) expression and tumor mutational burden, their performance may not be optimal and bring some practical issues including technical challenges and imperfect efficiency in accurately predicting ICIs responses^4^. Aside from the molecular biomarkers related to tumor genomics and tumor microenvironment, a growing body of research supports the potential importance of host-related characteristics such as psychological factors in cancer management^5,6^. Emotional distress (ED) refers to negative states or feelings that arise as a reaction to stressful stressors^7,8^. ED is commonly discerned from hallmarked symptoms of depression and anxiety, which can be assessed through standardized questionnaires^8–10^. Health challenges, such as a cancer diagnosis, can trigger ED, which is highly prevalent among patients with cancer. For example, individuals with lung cancer encounter symptoms of depression and/or anxiety with reported prevalence rates spanning from 33.0% to 77.2% (refs. ^11–14^). The incidence rate of ED is four times higher among patients with cancer than in the general population^5,15^.

Sustained ED activates the hypothalamic–pituitary–adrenal (HPA) axis and the sympathetic nervous system, which leads to the secretion of stress hormones, including glucocorticoids, and adrenergic mediators (such as epinephrine and norepinephrine)^16,17^. Chronic stress, a response to exposure to long-term and repeated stressors, impairs immune system functioning by consistently activating the HPA axis and the sympathetic nervous system^18,19^. Preclinical studies have demonstrated that distress-induced HPA axis activation and subsequent glucocorticoid release elicit anti-inflammatory effects, including the apoptosis of T lymphocytes and neutrophils^19^. The consistent release of endogenous glucocorticoids also inhibits antigen presentation, further undermining systemic immunological function^19^. Numerous animal studies have shown that exposure to chronic stress causes depression-like and anxiety-like symptoms in mice, which facilitates tumor progression and metastasis^16,20,21^. Stress hormones can directly affect the malignant molecular characteristics of tumor cells^16,22,23^. Preclinical studies have indicated that ED also plays a potential role in fostering the development of an immunosuppressive tumor microenvironment^16,24,25^. Exposure to chronic stress can reduce the proportion and dampen the functions of CD8^+^ T cells and natural killer cells, while also increasing the infiltration of immunosuppressive cells such as regulatory T cells and myeloid-derived suppressor cells^24,26,27^.

ED is also closely correlated with prognosis and survival outcomes in patients with cancer, as those experiencing depression and/or anxiety symptoms have elevated risks of recurrence and heightened mortality rates^14,28^. However, the screening and monitoring of ED are often disregarded and are not included in current medical routines^16^. With immunotherapy emerging as the cornerstone of NSCLC treatment, it is crucial to investigate the association between ED and the efficacy of ICIs. In murine tumor models, preliminary evidence indicates that chronic stress can impair the response to programmed cell death-1 (PD-1)/PD-L1 blockade^26,29,30^. However, there is limited clinical evidence to support a relationship between ED and the efficacy of ICIs, highlighting the necessity for prospective clinical studies.

To investigate this, we initiated a prospective observational study (STRESS-LUNG) with four cohorts. These cohorts aimed to investigate association of ED and lung cancer treatment and progression, including the efficacy of first-line treatment of ICIs in advanced NSCLC (cohort 1/STRESS-LUNG-1), the efficacy of first-line treatment of small cell lung cancer (cohort 2), the efficacy of neoadjuvant therapy with ICIs in resectable NSCLC (cohort 3), and cancer progression and prognosis in early-stage NSCLC receiving radical surgery (cohort 4). In this Article, we report the results for cohort 1 (STRESS-LUNG-1).

## Results

### Study design and patient baseline characteristics

**Extended Data Fig. 1:**
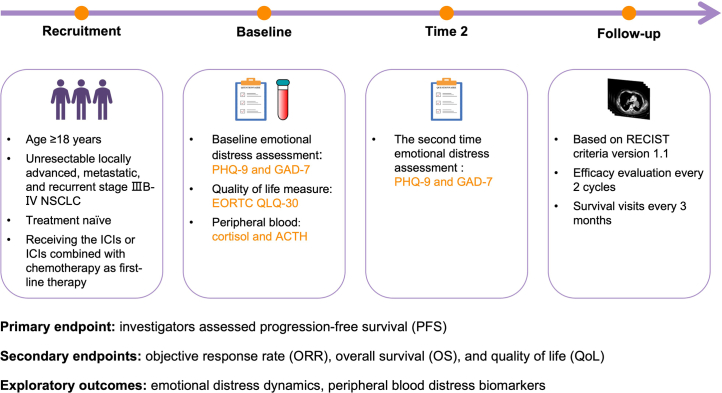
Study design of STRESS-LUNG-1. Stage IIIB-IV NSCLC patients who are about to receiving ICIs or combination therapy with chemotherapy as first-line treatment are screened for enrollment in this study. Emotional distress at baseline is assessed using the PHQ-9 and GAD-7 scales, while QoL is evaluated based on the EORTC QLQ-C30 questionnaire; A second assessment (Time 2) of emotional distress is conducted during follow-up. The efficacy of ICIs is evaluated every two cycles. Abbreviations: NSCLC, non-small-cell lung cancer; ICIs, immune checkpoint inhibitors; PHQ-9, Patient Health Questionnaire-9; GAD-7, Generalized anxiety disorder 7-item; EORTC QLQ-C30, European organization for research and treatment of cancer core quality of life questionnaire; ACTH, adrenocorticotropic hormone; RECIST, Response Evaluation Criteria in Solid Tumors.

This is a prospective observational study to analyze the association between ED and the efficacy of ICIs in advanced NSCLC. The primary endpoint is investigator-assessed progression-free survival (PFS). The secondary endpoints are objective response rate (ORR), overall survival (OS) and quality of life (QoL). The exploratory outcomes are the dynamic changes of ED during treatments, as well as peripheral blood ED biomarkers in the potential correlation between ED and survival outcomes. The study design is shown in Extended Data Fig. 1.

**Fig. 1:**
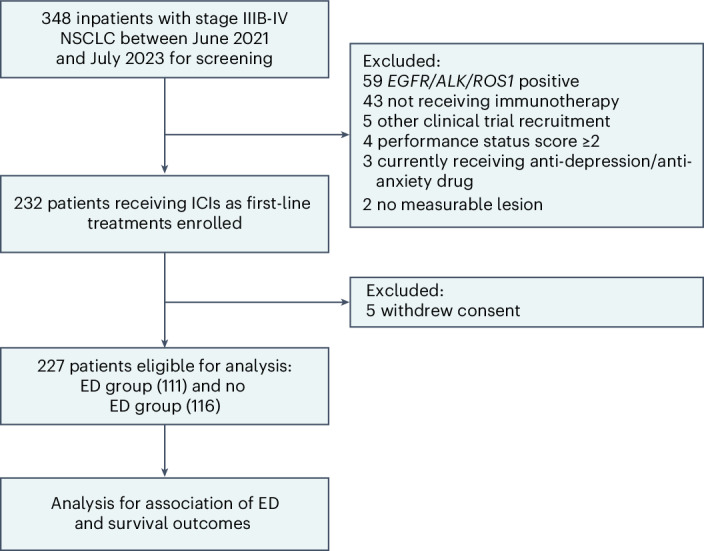
Flowchart diagram of STRESS-LUNG-1. The data cutoff was 30 November 2023.

As of 31 July 2023, 348 patients were screened, of whom 116 patients (33.3%) were excluded before enrollment; the main reasons for exclusion were epidermal growth factor receptor (*EGFR*)/ALK receptor tyrosine kinase (*ALK*)/ROS proto-oncogene 1, receptor tyrosine kinase (*ROS1*) positive, not receiving ICI treatment, and performance status (PS) score ≥2. A total of 227 patients with NSCLC were ultimately included for analysis in the study (Fig. 1). Most of the patients were male (92.5%), were diagnosed with stage IV NSCLC (58.1%), had a PS score of 1 (81.1%), were aged below 65 years (56.8%), were smokers (87.2%) and had lung squamous carcinoma (61.7%). The majority of patients had a positive expression of PD-L1 (71.8%), were receiving ICI combination treatments (94.7%), showed absence of brain/liver metastasis (86.3%), had no comorbidities of hypertension and diabetes (66.5%), had a body mass index (BMI) below 24 kg m^−^^2^ (67.0%) and had a neutrophil-to-lymphocyte ratio (NLR) below 5 (78.0%). The remaining demographic characteristics, including marital status, employment information, monthly income, caregiver and residence, are summarized in Table 1.

**Table 1:** Baseline demographic and clinical characteristics

Univariates	Total (*n* = 227)	No ED (*n* = 116)	ED (*n* = 111)	*P* value
**Age, years, median (IQR)**	63 (57–69)	63.5 (57–68)	63 (57–69)	
**Age, years,** ***n*** **(%)**				0.983
<65	129 (56.8)	66 (56.9)	63 (56.8)	
≥65	98 (43.2)	50 (43.1)	48 (43.2)	
**Sex,** ***n*** **(%)**				0.018
Male	210 (92.5)	112 (96.6)	98 (88.3)	
Female	17 (7.5)	4 (3.4)	13 (11.7)	
**PS,** ***n*** **(%)**				0.305
0	43 (18.9)	25 (21.6)	18 (16.2)	
1	184 (81.1)	91 (78.4)	93 (83.8)	
**Smoking status,** ***n*** **(%)**				0.129
Never smoked	29 (12.8)	11 (9.5)	18 (16.2)	
Current or former smokers	198 (87.2)	105 (90.5)	93 (83.8)	
**Smoking index,** ***n*** **(%)**				0.259
<400	52 (22.9)	23 (19.8)	29 (26.1)	
≥400	175 (77.1)	93 (80.2)	82 (73.9)	
**Histologic type,** ***n*** **(%)**				0.004
Squamous	140 (61.7)	82 (70.7)	58 (52.3)	
Nonsquamous	87 (38.3)	34 (29.3)	53 (47.7)	
**Disease stage,** ***n*** **(%)**				0.082
IIIB-C	95 (41.9)	55 (47.4)	40 (36.0)	
IV	132 (58.1)	61 (52.6)	71 (64.0)	
**PD-L1 expression,** ***n*** **(%)**				0.768
<1%	43 (18.9)	21 (18.1)	22 (19.8)	
1–49%	96 (42.3)	53 (45.7)	43 (38.7)	
≥50%	67 (29.5)	32 (27.6)	35 (31.5)	
Not detected	21 (9.3)	10 (8.6)	11 (9.9)	
**Treatment,** ***n*** **(%)**				0.206
Monotherapy	12 (5.3)	4 (3.4)	8 (7.2)	
Combination	215 (94.7)	112 (96.6)	103 (92.8)	
**No. metastatic organ sites,** ***n*** **(%)**				0.158
<2 sites	169 (74.4)	91 (78.4)	78 (70.3)	
≥2 sites	58 (25.6)	25 (21.6)	33 (29.7)	
**Brian/liver metastases,** ***n*** **(%)**				0.476
No	196 (86.3)	102 (87.9)	94 (84.7)	
Yes	31 (13.7)	14 (12.1)	17 (15.3)	
**Hypertension/diabetes,** ***n*** **(%)**				0.146
No	151 (66.5)	72 (62.1)	79 (71.2)	
Yes	76 (33.5)	44 (37.9)	32 (28.8)	
**BMI (kg** **m**^**−2**^**),** ***n*** **(%)**				0.109
<24	152 (67.0)	72 (62.1)	80 (72.1)	
≥24	75 (33.0)	44 (37.9)	31 (27.9)	
**NLR**				0.075
<5	177 (78.0)	96 (82.8)	81 (73.0)	
≥5	50 (22.0)	20 (17.2)	30 (27.0)	
**Marital status,** ***n*** **(%)**				0.717^a^
Married	220 (96.9)	113 (97.4)	107 (96.4)	
Divorced/widowed	7 (3.1)	3 (2.6)	4 (3.6)	
**Job status,** ***n*** **(%)**				0.482
Unemployed	103 (45.4)	50 (43.1)	53 (47.7)	
Employed	124 (54.6)	66 (56.9)	58 (52.3)	
**Caregiver,** ***n*** **(%)**				0.121
Spouse	121 (53.3)	56 (48.3)	65 (58.6)	
Children and others	106 (46.7)	60 (51.7)	46 (41.4)	
**Residence,** ***n*** **(%)**				0.073
Country	154 (67.8)	85 (73.3)	69 (62.2)	
City and town	73 (32.2)	31 (26.7)	42 (37.8)	
**Monthly income (CNY),** ***n*** **(%)**				0.619
<5,000	173 (76.2)	90 (77.6)	83 (74.8)	
≥5,000	54 (23.8)	26 (22.4)	28 (25.2)	
**Educational level,** ***n*** **(%)**				
<High school	158 (69.6)	84 (72.4)	74 (66.7)	0.347
≥High school	69 (30.4)	32 (27.6)	37 (33.3)	

^a^Proportion comparisons (marital status) analyses were calculated using a two-sided Fisher’s exact test. Proportion comparisons of the other characteristics were calculated using a two-sided *χ*^2^ test. CNY, Chinese Yuan.

**Extended Data Fig. 2:**
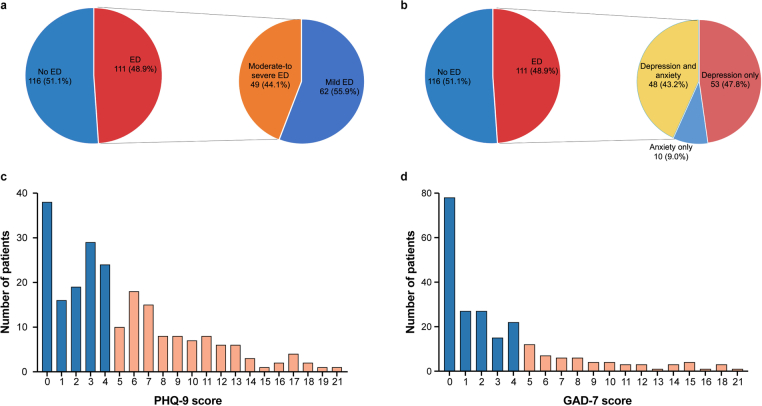
The distribution of emotional distress. **a**, Distribution of severity of emotional distress; **b**, Distribution of emotional distress, including depression and anxiety symptoms; **c**, Number of patients of each PHQ-9 score; **d**, Number of patients of each GAD-7 score; Abbreviations: ED, emotional distress. PHQ-9, Patient Health Questionnaire-9; GAD-7, Generalized anxiety disorder 7-item.

The median duration from pathological diagnosis to evaluation of ED was 16 days (interquartile range (IQR) 9–25 days). At the baseline assessment, 48.9% of patients (111/227) were found to be experiencing ED, which included 62 patients (55.9%) with mild ED and 49 patients (44.1%) with moderate-to-severe ED (Extended Data Fig. 2a). This ED group consists of 53 patients (47.8%) exhibiting depressive symptoms only, 10 patients (9.0%) with anxiety symptoms only and 48 patients (43.2%) with depressive and anxiety symptoms synchronously (Extended Data Fig. 2b). The number of patients for each score of Patient Health Questionnaire-9 (PHQ-9) and Generalized Anxiety Disorder 7-item (GAD-7) is shown in Extended Data Fig. 2c,d. Patients in the ED group versus the no ED group were more likely to have a higher proportion of females (13 of 111 (11.7%) versus 4 of 116 (3.4%); *P* = 0.018) and individias with nonsquamous pathology (53 of 111 (47.7%) versus 34 of 116 (29.3%); *P* = 0.004). The remaining baseline characteristics, such as PS score, disease stage, tumor PD-L1 expression and patterns of the ICI treatment and pathology, were well balanced between the ED and no ED groups. The multivariable logistic regression analysis for estimating the likelihood of ED showed similar results. Female individuals and patients with nonsquamous pathology showed high likelihood of ED, while other factors were not found to be associated with ED (Extended Data Table 1).

### Primary endpoint analysis

**Fig. 2:**
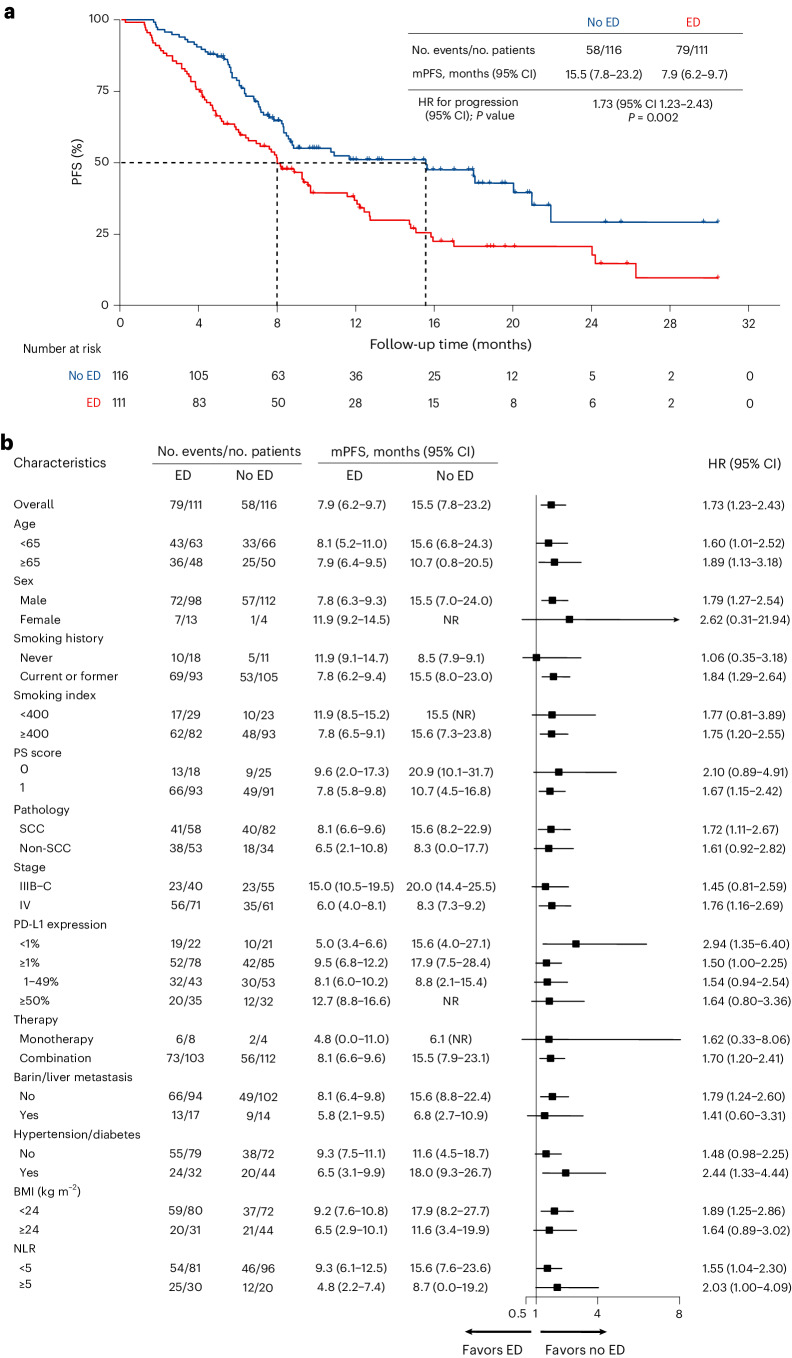
Kaplan–Meier curve and subgroup analysis of investigator-assessed PFS by baseline ED. **a**, Investigator-assessed PFS in the ED and no ED group. *P* values were calculated using a two-sided log-rank test. The HR and the corresponding 95% CI were calculated using a Cox proportional-hazards regression. **b**, The forest plots show HR and 95% CI of the subgroup analysis of PFS. Data are presented as the HR with error bars showing 95% CI. mPFS, median PFS; NR, not reached; SCC, squamous carcinoma.

The data cutoff was 30 November 2023. The median follow-up time was 16.0 months (95% confidence interval (CI) 14.0 to 18.0), and a total of 137 PFS events were observed. The median PFS for the overall population was 9.2 months (95% CI 7.0 to 11.4). The median PFS was 7.9 months (95% CI 6.2 to 9.7) in the ED group and 15.5 months (95% CI 7.8 to 23.2) in the no ED group (Fig. 2a). The median PFS was significantly shorter in the ED group than in the no ED group (hazard ratio (HR) 1.73, 95% CI 1.23 to 2.43; *P* = 0.002). Moreover, this consistent negative association between ED and PFS was evident across various subgroups (Fig. 2b), including the subgroups based on age (<65 years versus ≥65 years), PD-L1 expression (negative versus positive), NLR (<5 versus ≥5), male, smokers, lung squamous, stage IV, combination treatments and absence of brain/liver metastasis, among others. On multivariable Cox regression analysis, ED (HR 1.63, 95% CI 1.15 to 2.31; *P* = 0.006) and stage IV disease (HR 1.72, 95% CI 1.13 to 2.63; *P* = 0.012) emerged as independent predictors for worse PFS. On the contrary, PD-L1 expression ≥50% (HR 0.42, 95% CI 0.24 to 0.72; *P* = 0.002) was identified as a favorable predictor for PFS.

**Fig. 3:**
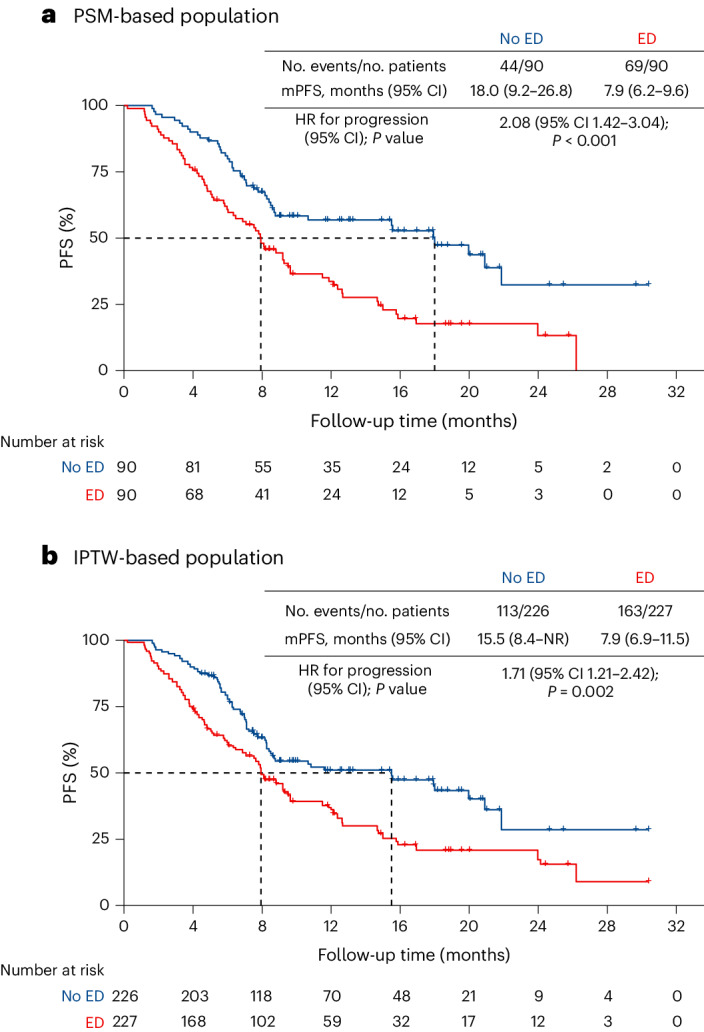
Kaplan–Meier curve of investigator-assessed PFS by baseline ED after propensity score analyses. **a**, Population based on PSM analysis. The exact *P* value was 0.0002. **b**, Population based on IPTW. In **a** and **b**, the final covariates were sex and pathology for PSM and IPTW, which were independently associated with ED on the basis of the multivariable logistic regression model. *P* values were calculated using a two-sided log-rank test. No adjustment was made for multiple comparisons. mPFS, median PFS.

The statistical methods of propensity score matching (PSM) and inverse probability of treatment weighting (IPTW) were performed to support the veracity of the associations (Table 2). There were 180 patients after PSM with well-balanced baseline characteristics (Extended Data Table 2). A total of 113 PFS events were observed. The median PFS was 7.9 months (95% CI 6.2 to 9.6) in the ED group and 18.0 months (95% CI 9.2 to 26.8) in the no ED group (Fig. 3a). The median PFS was significantly shorter in the ED group than in the no ED group (HR 2.08; 95% CI 1.42 to 3.04; *P* < 0.001). Furthermore, the IPTW analysis also confirmed this finding, demonstrating that the median PFS was significantly shorter in the ED group compared with the no ED group (HR 1.71; 95% CI 1.21 to 2.42; *P* = 0.002, Fig. 3b).

**Table 2:** Associations between ED and clinical efficacy of ICI

Endpoint	No ED (*n* = 116)	ED (*n* = 111)
**Primary endpoint**		
**PFS**		
No. events/no. patients at risk (%)	58/116 (50.0%)	79/111 (71.2%)
Cox proportional-hazards regression—HR (95% CI)	1.73 (1.23 to 2.43)	
Multivariable Cox regression—HR (95% CI)	1.63 (1.15 to 2.31)	
Propensity score analyses—HR (95% CI)		
With matching	2.08 (1.42 to 3.04)	
With inverse probability weighting	1.71 (1.21 to 2.42)	
**Secondary endpoints**		
**Type of response: no. (%)**		
Complete response	1 (0.9)	0 (0.0)
Partial response	71 (61.2)	52 (46.8)
Stable disease	36 (31.0)	42 (37.8)
Progression	2 (1.7)	9 (8.1)
Death	2 (1.7)	6 (5.4)
Could not be evaluated	4 (3.5)	2 (1.8)
**ORR: percentage of patients (95% CI)**	62.1 (53.2 to 70.9)	46.8 (37.6 to 56.1)
**Depth of response: % (range)**	−39.2 (−100.0 to 31.4)	−28.2 (−100.0 to 59.0)
**OS**		
1-Year rate (95% CI)	80.8% (70.9 to 87.6)	70.4% (60.3 to 78.4)
2-Year rate (95% CI)	64.9% (49.4 to 76.7)	46.5% (32.8 to 59.1)
Cox proportional-hazards regression—HR (95% CI)	1.82 (1.12 to 2.97)

**Extended Data Fig. 3:**
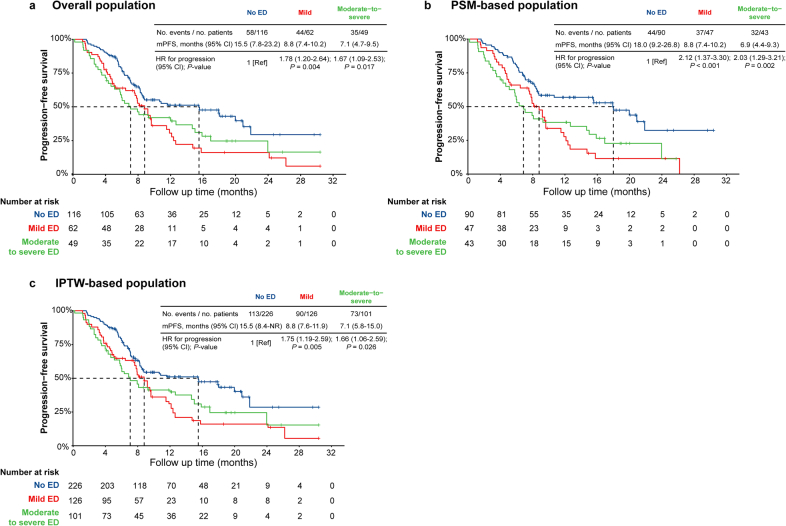
The associations between severity of emotional distress and progression-free survival. **a**, The overall population. **b**, PSM-based population; The exact *P* value was 0.0008 in comparison between no emotional distress (ED) group and mild ED group. **c**, IPTW-based population. **a**–**c**, *P* values were calculated using the two-sided log-rank test. **b**, **c**, The final covariates were sex and pathology for PSM and IPTW, which were independently associated with emotional distress based on multivariable logistic regression model. No adjustment was made for multiple comparisons. Abbreviations: ED, emotional distress; PSM, propensity score matching; IPTW, inverse probability of treatment weighting; HR, hazard ratio; CI, confidence interval; Ref, reference.

We further performed a sensitivity analysis between the severity of ED and PFS. The median PFS in the mild ED and moderate-to-severe ED groups was 8.8 months (95% CI 7.4 to 10.2) and 7.1 months (95% CI 4.7 to 9.5), respectively (Extended Data Fig. 3a). Compared with the no ED group, the mild ED group (HR 1.78, 95% CI 1.20 to 2.64; *P* = 0.004) and the moderate-to-severe ED group (HR 1.67, 95% CI 1.09 to 2.53; *P* = 0.017) exhibited a significantly increased risk of disease progression. Similar findings of shorter median PFS in the mild ED and moderate-to-severe ED groups were observed using the PSM (Extended Data Fig. 3b) and IPTW analysis (Extended Data Fig. 3c). In addition, we analyzed the role of depression and anxiety symptoms alone in diminishing the efficacy of ICIs. The depression symptoms were associated with shorter median PFS (HR 1.71, 95% CI 1.22 to 2.40; *P* = 0.002), as were the anxiety symptoms (HR 1.41, 95% CI 0.97 to 2.04; *P* = 0.070). We conducted a post hoc analysis to assess the association between ED and PFS using the alternative reported cutoff score of ‘a sum of PHQ-9 and GAD-7 scores ≥10’ (ref. ^31^), which indicated that the ED was associated with inferior PFS (HR 1.51; 95% CI 1.07 to 2.13; *P* = 0.020).

### Secondary clinical efficacy endpoint analysis

**Extended Data Fig. 4:**
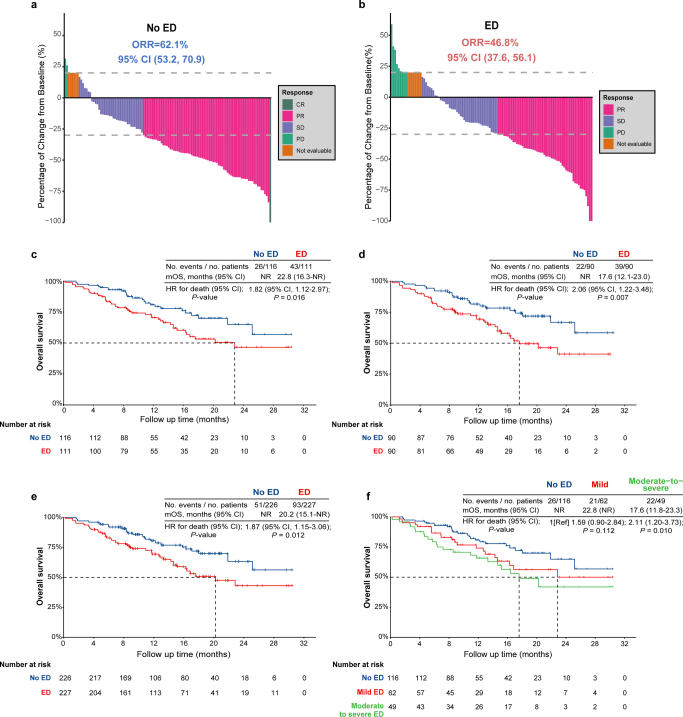
The associations between emotional distress, objective response rate and overall survival. **a**, Waterfall plots for best percentage change in target lesion size for no emotional distress (ED) group; **b**, Waterfall plots for best percentage change in target lesion size for ED group. **c**, The overall survival of ED and no ED group in overall population; **d**, The overall survival of ED and no ED group in PSM-based population; **e**, The overall survival of ED and no ED group in IPTW-based population; **f**, The association between severity of emotional distress and overall survival in the overall population. **d**, **e**, The final covariates were sex and pathology for PSM and IPTW, which were independently associated with emotional distress based on multivariable logistic regression model. **c**–**f**, *P* values were calculated using the two-sided log-rank test. No adjustment was made for multiple comparisons. Abbreviations: ED, emotional distress; ORR, objective response rate; PSM, propensity score matching; IPTW, inverse probability of treatment weighting; HR, hazard ratio; CI, confidence interval; Ref, reference.

The ORR was 54.6% (95% CI 48.1 to 61.1) in the overall population. The ORR was 46.8% (95% CI 37.6 to 56.1) in the ED group and 62.1% (95% CI 53.2 to 70.9) in the no ED group, indicating that the ED group had a significantly lower ORR (odds ratio (OR) 0.54; 95% CI 0.32 to 0.91; *P* = 0.022). The ORR for the mild ED and moderate-to-severe ED groups was 50.0% (95% CI 37.6 to 62.4) and 42.9% (95% CI 29.0 to 56.7), respectively. The mild ED group (OR 0.61; 95% CI 0.33 to 1.14; *P* = 0.121) and the moderate-to-severe ED group (OR 0.46; 95% CI 0.23 to 0.90; *P* = 0.024) showed a lower ORR compared with the no ED group. The median best percentage change in target lesion size (maximum decrease from baseline, or minimum increase from baseline in the absence of a decrease) was −28.2% (range −100 to 59.0) in the ED group versus −39.2% (range −100 to 31.4) in the no ED group (*P* = 0.008). Detailed information on the depth of response in the two groups is shown in Extended Data Fig. 4a,b.

A total of 69 OS events were observed, and the median OS in the overall population was not yet mature. Compared with the no ED group, the ED group had a higher risk of death from any cause (HR 1.82, 95% CI 1.12 to 2.97; *P* = 0.016, Extended Data Fig. 4c), with a lower 1-year OS rate (70.4% versus 80.8%) and 2-year OS rate (46.5% versus 64.9%). A total of 61 events occurred after PSM analysis, with the ED group having a higher risk of death from any cause (HR 2.06, 95% CI 1.22 to 3.48; *P* = 0.007, Extended Data Fig. 4d). After IPTW analysis, the median OS was significantly shorter in the ED group than in the no ED group (HR 1.87, 95% CI 1.15 to 3.06; *P* = 0.012, Extended Data Fig. 4e). The 1-year survival rates of the mild ED group and the moderate-to-severe ED group were 74.1% (95% CI 59.8 to 83.9) and 65.8% (95% CI 50.2 to 77.5), respectively. The 2-year survival rates were 50.0% (95% CI 31.2 to 66.2) and 41.9% (95% CI 23.1 to 59.7), respectively. Compared with the no ED group, the mild ED group (HR 1.59, 95% CI 0.90 to 2.84; *P* = 0.112) and the moderate-to-severe ED group (HR 2.11, 95% CI 1.20 to 3.73; *P* = 0.010) had an increased risk of OS events (Extended Data Fig. 4f).

### QoL analysis

A total of 206 patients completed a baseline assessment of QoL, revealing that the ED group exhibited worse outcomes on most subscales of QoL compared with the no ED group. Firstly, the ED group displayed lower global health scores as well as lower scores in individual functional domains including physical functioning, role functioning, emotional functioning, cognitive functioning and social functioning. Additionally, the ED group reported more symptoms of fatigue, pain, dyspnea, insomnia, loss of appetite, and constipation, along with experiencing economic difficulties, except for nausea, vomiting and diarrhea (Extended Data Table 3).

### Dynamic changes in ED

**Fig. 4:**
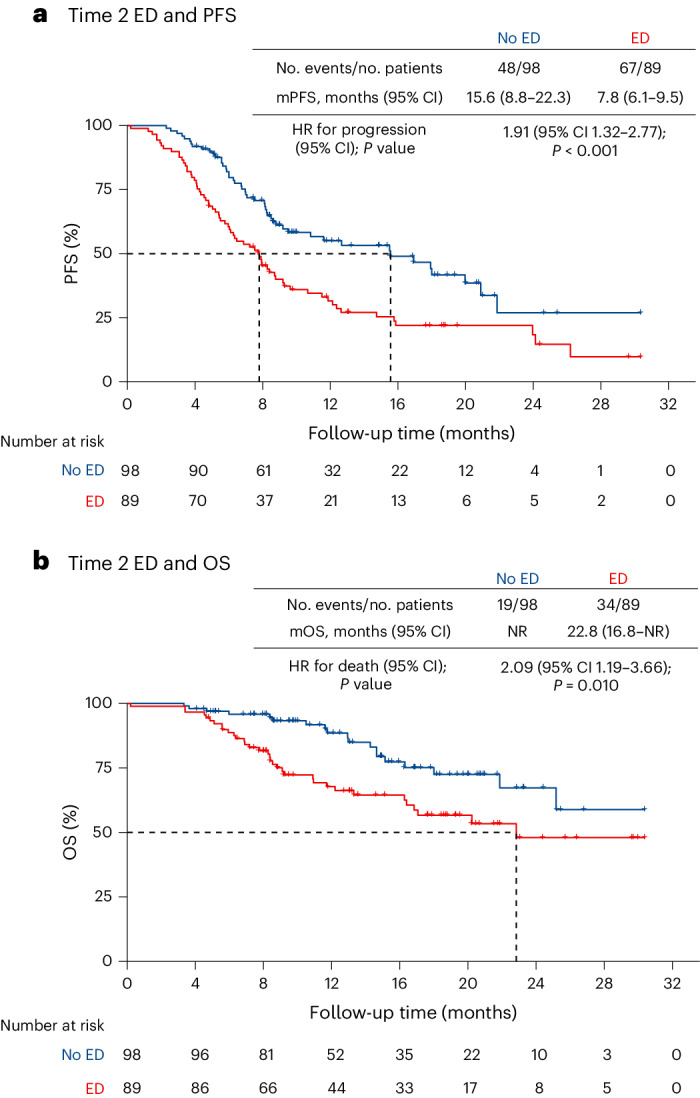
Survival analyses by Time 2 ED. **a**, The PFS analysis in the ED and no ED groups by Time 2 assessments. The exact *P* value was 0.0007. **b**, The OS analysis in the ED and no ED groups by Time 2 assessments. In **a** and **b**, *P* values were calculated using a two-sided log-rank test. mPFS, median PFS; mOS, median OS; NR, not reached.

**Extended Data Fig. 5:**
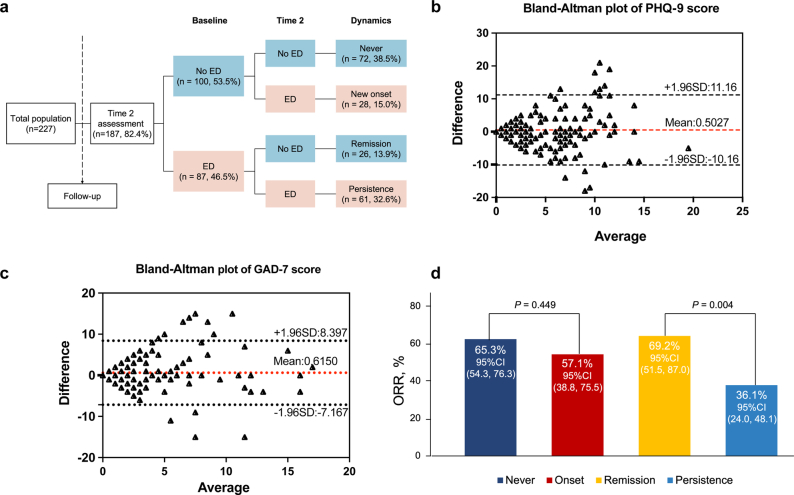
Emotional distress dynamics from baseline to follow-up. **a**, Longitudinal changes of emotional distress; **b**, The consistent analysis of PHQ-9 score between baseline and Time 2 assessments (n = 187); **c**, The consistent analysis of GAD-7 score between baseline and Time 2 assessments (n = 187); **d**, The associations between emotional distress dynamics and objective response rate*, P* values were calculated using the two-sided χ^2^ test. Abbreviations: ED, emotional distress; PHQ-9, Patient Health Questionnaire-9; GAD-7, Generalized anxiety disorder 7-item; SD, standard deviation; ORR, objective response rate.

We performed an exploratory analysis of the dynamic changes of ED in the follow-up. A total of 187 patients (82.4%) completed both baseline and Time 2 ED assessment on PHQ-9 and GAD-7, with a median interval of 2.9 months (IQR 1.9 to 5.0). In the baseline no ED group, 72 patients (38.5%) had ‘never ED’ and 28 patients (15.0%) had ‘ED new onset’. In the baseline ED group, 61 patients (32.6%) presented ‘ED persistently’ and 26 patients (13.9%) presented ‘ED remission’ (Extended Data Fig. 5a). The ED state of 133 patients (71.1%) remained consistent. A good correlation coefficient in PHQ-9 and GAD-7 scores is shown between the baseline and the Time 2 assessment (Extended Data Fig. 5b,c). A total of 115 PFS events occurred, with a median PFS of 9.2 months (95% CI 7.0 to 11.4) in patients accomplishing the Time 2 assessment. Based on the Time 2 ED state, the median PFS was 7.8 months (95% CI 6.1 to 9.5) in the ED group and 15.6 months (95% CI 8.8 to 22.3) in the no ED group. The ED group had a shorter PFS (HR 1.91, 95% CI 1.32 to 2.77; *P* < 0.001, Fig. 4a) and a higher risk of OS events (HR 2.09, 95% CI 1.19 to 3.66; *P* = 0.010, Fig. 4b).

**Extended Data Fig. 6:**
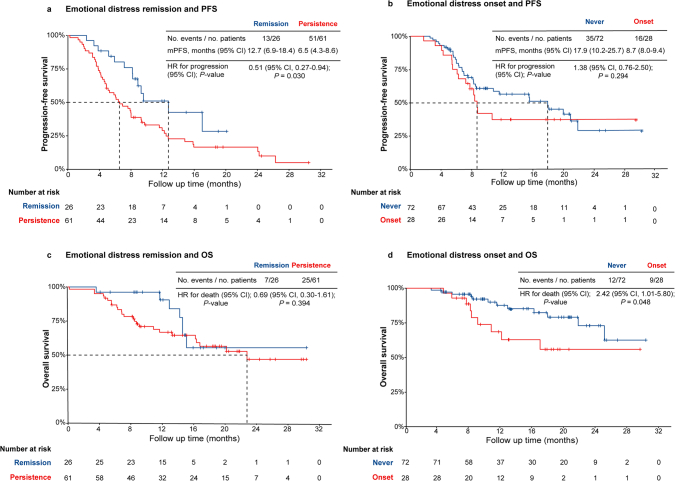
The associations between emotional distress dynamics and survival outcomes. **a**, The progression-free survival analysis in emotional distress (ED) remission and persistence subgroups; **b**, The progression-free survival analysis in never and onset subgroups; **c**, The overall survival analysis in remission and persistence subgroups; **d**, The overall survival analysis in never and onset subgroups. **a**–**d**, *P* values were calculated using the two-sided log-rank test. Abbreviations: ED, emotional distress; PFS, progression-free survival; OS, overall survival; HR, hazard ratio; CI, confidence interval.

In the four ED dynamic subgroups for never, onset, remission and persistence, the ORRs were 65.3%, 57.1%, 69.2% and 36.1%, respectively (Extended Data Fig. 5d). ED remission was associated with improved ORR compared with the persistence group (*P* = 0.004). The ORR between the never group and the onset group had no significant difference (*P* = 0.449). The median PFS was 17.9 months (95% CI 10.2 to 25.7), 8.7 months (95% CI 8.0 to 9.4), 12.7 months (95% CI 6.9 to 18.4) and 6.5 months (95% CI 4.3 to 8.6) for never, onset, remission and persistence group, respectively. Compared with the persistence group, the remission group had a longer median PFS (HR 0.51, 95% CI 0.27 to 0.94; *P* = 0.030, Extended Data Fig. 6a). Compared with the never group, the onset group did not have increased risk of PFS events (HR 1.38, 95% CI 0.76 to 2.50; *P* = 0.294, Extended Data Fig. 6b). A total of 53 OS events occurred. There was no difference in OS between the remission group and the persistence group (HR 0.69, 95% CI 0.30 to 1.61; *P* = 0.394, Extended Data Fig. 6c). Compared with the never group, the onset group had an increased risk of OS events (HR 2.42, 95% CI 1.01 to 5.80; *P* = 0.048, Extended Data Fig. 6d).

### Peripheral blood distress biomarkers

**Extended Data Fig. 7:**
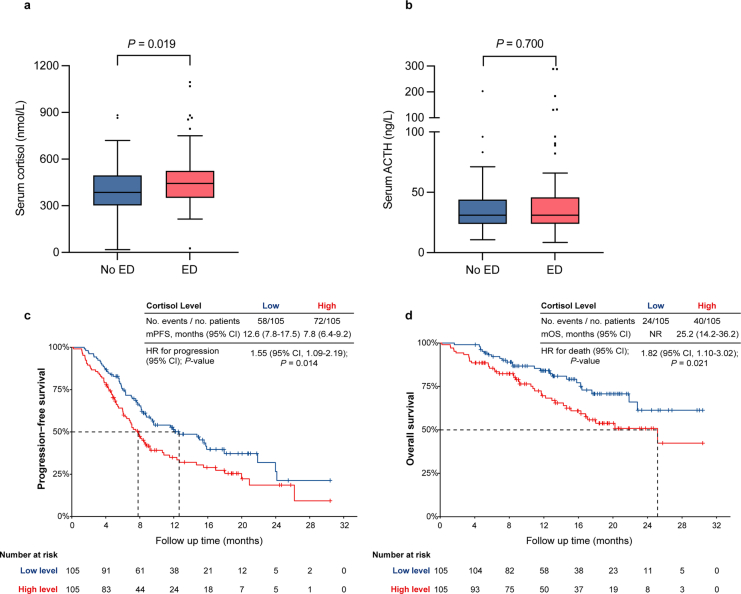
The association of peripheral blood stress hormones with emotional distress and clinical outcomes. **a**, Serum cortisol level in emotional distress (ED, n = 107) and no emotional distress (no ED, n = 103) groups; **b**, Serum ACTH level in ED (n = 107) and no ED (n = 103) groups; **c**, The progression-free survival analysis in different cortisol levels groups; **d**, The overall survival analysis in different cortisol levels groups. **a**, **b**. For each boxplot, the box is presented as median (interquartile range), whiskers extend from the hinges to the largest (or smallest) value no further than 1.5 interquartile range from the hinge. *P*-values were calculated using a two-sided nonparametric Mann–Whitney U test. **c**, **d**. *P* values were calculated using the two-sided log-rank test. Abbreviations: HR, hazard ratio; CI, confidence interval.

We performed an exploratory analysis of peripheral blood distress biomarkers including stress hormones. A total of 210 patients were tested for serum cortisol and adrenocorticotropic hormone (ACTH) at baseline. The ED group had higher serum cortisol (443.4 versus 386.0 nmol l^−1^, *P* = 0.019, Extended Data Fig. 7a) compared with the no ED group. There were no significant differences in serum ACTH (31.0 versus 31.1 ng l^−1^, *P* = 0.700, Extended Data Fig. 7b) between the ED and no ED groups. As the cutoff of median cortisol level, the median PFS was 7.8 months (95% CI 6.4 to 9.2) in the high cortisol level group and 12.6 months (95% CI 7.8 to 17.5) in the low cortisol level group. The group with higher levels of cortisol had shorter median PFS (HR 1.55, 95% CI 1.09 to 2.19, *P* = 0.014, Extended Data Fig. 7c). Patients with higher cortisol levels had a lower trend of ORR (51.4% versus 60.0%, *P* = 0.211) and a higher risk of OS events (HR 1.82, 95% CI 1.10 to 3.02, *P* = 0.021, Extended Data Fig. 7d). Serum ACTH levels did not show a significant association with the efficacy of ICIs in patients.

## Discussion

Patients with cancer often experience ED due to multiple stressors, including the diagnosis itself, the burden of symptoms, challenges in social relationships, and financial toxicity^13,32,33^. Most previous studies looking at the relationship between ED and clinical outcomes commonly with objectives on QoL and prognosis mostly derived results from retrospective clinical data in patients with cancer^14,28,34–38^. Few studies have explored the relationship between ED and the efficacy of immunotherapy. In this study, we found an association between ED and worse outcomes following treatment with ICIs in patients with advanced NSCLC. Notably, patients with ED demonstrated shorter median PFS, lower ORR and higher risk of OS events compared with the no ED group.

Preclinical studies provide the complementary evidence on the effect of ED and cancer progression and treatment efficacy. Neuroendocrine regulation is a classic mechanism explaining that how ED influences immunosurveillance and dampens the antitumor immune response^19,24^. Yang et al. demonstrated that ED caused by social defeat can elevate plasma cortisol levels and expression of Tsc22d3 on dendritic cells, which inhibited the activation of CD8^+^ T cells in sarcomatoid and colorectal cancers mice^26^. Standard-temperature-induced stress suppresses the functioning of CD8^+^ T cells by regulating the β2-adrenergic receptor signaling and undermines the PD-1 blockade responses in mice with melanoma and breast cancer^29^. Moreover, ED induced by acute restrains promotes the release of kisspeptin and activates the GPR54 signaling, which results in T cell dysfunction and facilitates the immune escape in mice with lung cancer^27^. In this study, we found that higher levels of cortisol were associated with worse survival. These findings indicated the potential role of neuroendocrine regulation in mediating the association between ED and the efficacy of ICIs.

Our study suggests that intervention strategies to alleviate ED may be a potential strategy to enhance the efficacy of ICIs. Preclinical studies have demonstrated that glucocorticoid receptor inhibitors and β-adrenergic receptor inhibitors (β-blockers) can potentially improve the efficacy of ICIs in tumor mouse models^26,39^. Nutritional and psychological support integrated into chemotherapy prolongs drug treatment duration and improves survival outcomes in patients with esophagogastric cancer^40^. Concomitant use with β-blockers was positively associated with better prognosis in patients with NSCLC receiving *EGFR* tyrosine kinase inhibitors^41^. Retrospective studies indicates that the concomitant use of β-blockers with ICIs was associated with better survival outcomes in patients with solid tumor^42,43^. A phase I trial showed that β-blockers combined with PD-1 inhibitors had promising efficacy in patients with advanced melanoma^44^. These findings collectively highlight the potential treatment response benefits of addressing ED. To this end, we have launched the BRIO study (ClinicalTrials.gov ID: NCT05979818) to investigate the efficacy and safety of β-blockers combined with ICIs and chemotherapy in first-line therapy for patients with advanced-stage NSCLC.

Recently, a post hoc analysis of a phase II PRADO trial aimed to investigate the correlation between pretreated ED, and the efficacy of neoadjuvant immunotherapy in patients with melanoma was reported^45^. Poll-Franse et al. observed that, in the baseline ED group, there were decreased major pathologic responses (46% versus 65%) and reduced 2-year relapse-free survival (74% versus 91%), compared with the no ED group^45^. However, this post hoc analysis warrants further validation, and our prospective cohort study lends support to the association between ED and ICI efficacy. Prospective cohort studies may be the most suitable method for examining the association, while randomized control trials are not feasible since ED is the natural response to stressors, which cannot be induced by intervention. Besides, there is a difference between the tools to measure ED. The PRADO trial used the emotional functioning extracted from the Treatment of Cancer Core Quality of Life Questionnaire (EORTC QLQ-C30), a scale of measuring QoL to estimate ED^45^. We utilized standardized screening scales of depression and anxiety symptoms scales to determine ED^46,47^. The EORTC QLQ-C30 holds advantages, given its widespread application in oncology clinical trials that provide sufficient data to analyze the correlation^48^. The combined assessment of PHQ-9 and GAD-7 enables the evaluation of affective and somatic symptoms associated with ED, as well as the quantification of the severity of depression and anxiety^46,47,49^. These two questionnaires would serve as screeners to measure symptoms of depression and anxiety, aiding in the determination regarding the further diagnostic procedures and psychological intervention treatments^50,51^.

In this cohort study, we meticulously considered various sociodemographic characteristics to figure out the factors that may affect the ED, including educational levels, financial situations and disease burdens^52,53^. We found that female patients and patients with nonsquamous pathology were more likely to experience ED, allowing us to identify susceptible groups for ED management. To alleviate the effect of confounding variables on ED and survival outcomes, we employed rigorous statistical methods, including PSM and IPTW analyses, which may enhance the veracity of the association^54^. In addition, in the subgroup analysis of PFS, we observed a negative association between ED and survival outcomes regardless of whether the patient had positive or negative PD-L1 expression. In the subgroup of females and monotherapy, we observed that the 95% CI of HR crosses 1, which might be attributed to the relatively small sample size. The lower percentage of female participants may be attributed to the fact that never-smoking females in China often have a higher rate of *EGFR*/*ALK*/*ROS1* gene positivity, and the distribution of sex in our study aligned with clinical studies in the Chinese population^55–57^. Nevertheless, the sex imbalance is one of the limitations of this study. Notably, our study delved into the severity of ED and its correlation with the efficacy of ICIs. Patients with higher severity of ED tended to experience worse survival outcomes including ORR and OS.

Utilizing biomarkers solely, such as tumor PD-L1 and tumor mutational burden, was not a fully satisfying tool to predict the response of ICIs^58^. The psychological dimension plays a potential role in cancer treatment. Therefore, the combination of predictive biomarkers and psychological factors may be beneficial. Importantly, our research demonstrates that the ED remained relatively consistent from baseline to follow-up, which was also supported by previous studies indicating that ED is a stable psychological parameter in patients with cancer^59,60^. Therefore, we proposed the concept of ‘psycho-biomarker’ to predict the efficacy of the PD-1/PD-L1 inhibitors in NSCLC. This approach broadens the potential predictive factors for immunotherapy outcomes and underscores the importance of addressing the psychological dimension in cancer care. In addition, our study serves as a reference for future clinical trials, which means the investigators should consider incorporating ED as a baseline characteristic when designing studies.

This study has several limitations. It is a single-center observational study, and the data of OS are not fully matured. While the study suggests an association between ED and reduced efficacy of ICIs in NSCLC, a causal link cannot be established. This will require further investigation in preclinical studies and in human cohort studies with more samples. We also acknowledge the possibility that potential confounders may be associated with outcomes. Another significant limitation is that most patients were male and all patients were Chinese. These results will therefore need validation in additional cohorts of female patients and differing ancestries. Various types of PD-1/PD-L1 inhibitor were included in this study, which could impact the clinical responses observed. Finally, utilizing a cutoff score of ‘PHQ-9 or GAD-7 ≥5’ for screening ED demonstrated great efficiency in predicting the efficacy of ICIs in NSCLC. However, it is worth noting that other cutoff scores (for example, a sum score ≥10) may also be relevant. It is important to incorporate gold-standard structured clinical interviews to explore potential cutoff scores and validate this approach. It is essential to investigate whether an examiner rating scale should be combined with patient self-rating scales to determine ED, and we will investigate this further in the following BRIO study.

In summary, our study has established an association between ED and diminished efficacy of ICIs in patients with advanced NSCLC. Moreover, this study provides real-world clinical insights into how ED correlates with the survival outcomes of patients with cancer receiving ICIs. The concept of a ‘psycho-biomarker’ may be relevant for not only NSCLC but also other cancer types in the future.

## Methods

### Study design

We initiated the STRESS-LUNG study to investigate the association between ED and the efficacy of cancer therapy and prognosis in lung cancer with four cohorts (ClinicalTrials.gov Identifier: NCT05477979). Cohort 1, referred to as the STRESS-LUNG-1 cohort (also named PORG2101), is a prospective observational cohort study to explore the association between ED and the efficacy of first-line treatment of ICIs in advanced NSCLC. The primary endpoint of the study is the investigator-assessed PFS. The secondary endpoints include the ORR, OS and QoL. Exploratory outcomes involve the analysis of ED dynamics and the relationships between peripheral blood distress biomarkers and the efficacy of ICI treatments. The design of the STRESS-LUNG study is available on ClinicalTrials.gov^61^. The study was registered on 26 July 2022. The primary endpoint of the STRESS-LUNG-1 cohort has one modification: the adjustment from ‘ORR and PFS’ to ‘PFS’. The main reason for the adjustment is that a single endpoint is more likely to clarify the scientific results of observational studies, while multiple-endpoint studies are relatively complex and require large sample sizes. Besides, the study identification was updated into the STRESS-LUNG study with four cohorts. We here report the results of cohort 1 (STRESS-LUNG-1), and the other three cohorts are ongoing.

### Participants

Eligible patients were screened and recruited from 1 June 2021 to 31 July 2023 at the Second Xiangya Hospital of Central South University. The inclusion criteria were as follows: (1) adults aged 18 years or older; (2) histologically confirmed diagnosis of NSCLC; (3) unresectable locally advanced, metastatic, or recurrent stage IIIB-IV based on AJCC TNM staging 8th edition; (4) Eastern Cooperative Oncology Group PS of 0–1; (5) treatment naive; (6) presence of at least one measurable lesion according to the Response Evaluation Criteria in Solid Tumors version 1.1 (RECIST v1.1); (7) receiving PD-1/PD-L1 inhibitor monotherapy or combination with chemotherapy as first-line therapy; (8) informed consent to participate in the study. Exclusion criteria were as follows: (1) *EGFR* mutations of ex19del and ex21 L858R, *ALK* fusion positive and *ROS1* fusion positive; (2) presence of other malignant tumors or malignant diseases within 3 years; (3) concurrent acute or chronic psychiatric disorders; (4) patients currently receiving anti-depression or anti-anxiety drug therapy; (5) prior participation in other clinical drug trials; (6) symptomatic brain metastases; (7) inability to complete psychological scale assessments. This study was approved by the Ethics Committee of the Second Xiangya Hospital of Central South University (2021073). Written informed consent was signed by all patients.

### Treatment procedures

The treatment protocol involving the use of PD-1 and PD-L1 inhibitors, such as pembrolizumab, atezolizumab, camrelizumab, sintilimab, tislelizumab, toripalimab and sugemalimab, was approved by China’s National Medical Products Administration for first-line therapy in advanced nonsquamous and squamous NSCLC. For patients with nonsquamous NSCLC, the chemotherapy regimen included pemetrexed and platinum. For squamous NSCLC, it included platinum and either paclitaxel, nab-paclitaxel or gemcitabine. This combination therapy was administered for durations of four to six cycles, followed by maintenance treatment with ICI monotherapy for squamous and ICIs and/or pemetrexed for nonsquamous. The maintenance therapy with ICIs continued for up to 2 years or until disease progression, death or unacceptable toxicity. The monotherapy with pembrolizumab was an alternative treatment option for patients with exhibiting tumor PD-L1 expression of ≥1%, as well as atezolizumab monotherapy for patients with exhibiting tumor PD-L1 expression of ≥50%. The maintenance phase for ICIs lasted up to 2 years or until disease progression, death or unacceptable toxicity.

### Data collection and evaluation

Baseline characteristics were documented in an electronic case report form, including information on sociodemographic and radiographic aspects evaluated up to the preceding month before immunotherapy initiation, as well as at two-cycle intervals until the final follow-up. We collected the sex information of participants determined by biological sex (female or male). Survival visits were performed every 3 months. PFS was defined as the duration between the date of initiation of ICIs and disease progression or death, whichever occurred first. The depth of response was quantified as the percentage representing the largest decrease from baseline or the smallest increase from baseline with no reduction, including all assessments before the progression or initiation of further anticancer therapy. ORR was determined by calculating the percentages of patients who exhibited a confirmed complete response, defined as the disappearance of all lesions and pathological lymph nodes with a reduction in short axis to <10 mm, and a partial response, characterized by at least a 30% decrease in the size of target lesions, based on RECIST v1.1 criteria^62^. OS refers to the duration between the date of initiation of ICIs and death from any cause. The data cutoff was 30 November 2023.

### Evaluation of ED

ED commonly refers to depression and/or anxiety symptoms^8–10,63^. PHQ-9 and GAD-7 were recommended in screening the depressive and anxiety symptoms in patients with cancer, respectively^8,64^.

PHQ-9 is a nine-item questionnaire, self-rated, validated scale for screening depression symptoms and measuring its severity rated on a scale of 0 (not at all), 1 (several days), 2 (more than half the days) or 3 (nearly every day). As a total score ranging from 0 to 27, the PHQ-9 score was divided into the following categories of increasing severity: none (0–4), mild (5–9), moderate (10–14), moderately severe (15–19) and severe (20–27)^46^. A PHQ-9 score of 5 or more suggests the presence of depression symptoms^46,65^. The severity of depressive symptoms in this study was classified into three subgroups according to the PHQ-9 score: no depression (0–4), mild depression (5–9) and moderate-to-severe depression (≥10). The PHQ-9 was a brief and valid tool in screening depression symptoms and the severity in individuals^66^. In addition, the PHQ-9 has been translated into Chinese and validated in clinical research, and the cutoff score of 5 demonstrated a favorable reliability and validity for screening depressive symptoms with a sensitivity of 0.91, specificity of 0.77 in chronic disease conditions, and a Cronbach’s *α* of 0.89 in patients with cancer^65,67,68^. The cutoff score and its severity of PHQ-9 had been extensively applied to screen symptoms of depression in studies involving patients with cancer^65,69^.

GAD-7 is a seven-item, self-rated, validated scale for screening anxiety symptoms and measuring its severity rated on a scale of 0 (not at all), 1 (several days), 2 (more than half the days) or 3 (nearly every day)^47^. With a total score ranging from 0 to 21, a GAD-7 score ≥5 suggests the presence of anxiety symptoms, and scores of 5, 10 and 15 represent mild, moderate and severe anxiety symptoms, respectively^70–72^. The severity of anxiety symptoms in this study was classified into three subgroups based on the GAD-7 scores: no anxiety (0–4), mild anxiety (5–9) and moderate-to-severe anxiety (≥10). GAD-7 was also translated into Chinese and had shown satisfactory validity and reliability for screening anxiety symptoms with a Cronbach’s *α* of 0.91 in patients with cancer, which was widely applied in clinical studies^47,65^.

The ED is determined by either PHQ-9 score ≥5 or GAD-7 score ≥5 according to validated thresholds in clinical practice^46,47,73,74^. Classifications of different severity of ED were based on PHQ-9 and/or GAD-7 scores. No ED was determined as both PHQ-9 and GAD-7 scores were 0 to 4; mild ED refers to mild depression and/or anxiety symptoms (either PHQ-9 or GAD-7 scores were 5 to 9); moderate-to-severe ED refers to moderate-to-severe depression and/or anxiety symptoms (either PHQ-9 or GAD-7 scores ≥10)^73,75^.

ED was measured at baseline before initiation of ICI treatment. The second time (Time 2) assessment of ED was conducted after at least two cycles of the administration of PD-1/PD-L1 inhibitors. Patients were classified into four subgroups based on dynamic changes of ED from baseline to follow-up (Time 2). Those without ED were defined as ‘never’, those with new ED onset at Time 2 were defined as ‘onset’, those with ED remission at Time 2 were defined as ‘remission’ and those with persistent ED from baseline to Time 2 were defined as ‘persistence’ (Extended Data Fig. 5a)^14^. The patients responded to each item of the questionnaires and completed self-administered scales after a thorough explanation of the scale’s purpose.

### Assessment of QoL

QoL was assessed using the European Organization for Research and Treatment of Cancer Core Quality of Life Questionnaire (EORTC QLQ-C30), a self-administered tool tailored for patients with cancer^76,77^. These 30 items comprise five multi-item functional domains (physical, role, emotional, cognitive and social), nine symptom domains (fatigue, nausea/vomiting, pain, dyspnea, insomnia, appetite loss, constipation, diarrhea and financial difficulties) and a global health status. Items 29 and 30 are divided into seven levels, and the remaining domains were four-point Likert scale, with scores ranging from 0 to 100. Higher scores in the global health and functional domains indicate a higher QoL. In contrast, higher scores in the symptom domains indicate a lower QoL.

### Stress hormone determination

Venous blood samples were uniformly collected from patients, commencing at 8:00 before the ICI treatment. The blood collection occurred following a 15-min supine rest period and a 12-h abstinence from alcohol, coffee and tobacco consumption. The blood samples were promptly centrifuged at 1,811*g* and 4 °C for 15 min after collection. Furthermore, data on serum cortisol and ACTH levels, obtained as part of routine clinical laboratory examinations before initiating ICI treatment, were collected. Serum cortisol and ACTH concentrations were determined utilizing the Siemens ADVIA Centaur XP immunoassay system and the Siemens IMMULITE 2000XPi immunoassay system, respectively.

### Statistical analysis

Categorical variables were described as frequencies and percentages. Normally distributed data were presented as mean ± standard deviation (SD), with paired or unpaired *t-*tests used for comparisons. Nonnormally distributed data were described by the median and IQR, and the Mann–Whitney *U* test and Wilcoxon test were used for comparisons. Proportion comparisons analyses utilized the *χ*^2^ test and Fisher’s exact test (for low-sample dichotomous data). The consistency of baseline and secondary ED states was assessed through Kappa analysis (for categorical data), and Bland–Altman analysis (for numerical data).

To evaluate the association between ED and primary and secondary outcomes, survival curves were estimated by using the Kaplan–Meier method and compared with the log-rank test. Cox proportional-hazards regression was used to estimate the association between ED and survival outcomes. Multivariable Cox regression was used to evaluate the clinical prognostic values, with variable selection with prior decisions by investigators (12 events per degree of freedom). PSM and IPTW methods were employed to balance covariate distributions between the ED and no ED groups^78,79^. Propensity scores for the likelihood of ED states were estimated using a multivariable logistic regression model that included demographic, clinicopathological and predetermined laboratory factors^80^. The final covariates were sex and pathology for PSM and IPTW, which were associated with ED. For the matching, a nearest-neighbor method with a 1:1 ratio (ED:no ED) and a caliper width of 0.2 were adopted. IPTW was used as an alternative approach to conduct outcome comparison between the ED and no ED groups^54^. In IPTW, each patient’s weight was calculated by inverting the probability of ED states. Sensitivity analysis was performed by using the variate of the severity of ED and analyzing its association with clinical efficacy outcomes including PFS, ORR and OS. We conducted a post hoc analysis to analyze the association between ED and PFS using alternative reported cutoff score of ‘a sum of PHQ-9 and GAD-7 scores ≥10’ (ref. ^31^). In addition, we utilized the Surv_cutpoint function of survminer package in R and a Kaplan–Meier curve log-rank test to determine the optimal cutoff score of PHQ-9 and GAD-7 for distinguishing the risk of disease progression (Supplementary Figs. 1–3). Statistical analyses were conducted on SPSS 24.0 (IBM), R (v. 4.2.1) and R Studio (v. 1.4.1717). The plots were generated using GraphPad (Prism 7) and R (v. 4.2.1), and R Studio (v. 1.4.1717).

### Reporting summary

Further information on research design is available in the Nature Portfolio Reporting Summary linked to this article.

## Online content

Any methods, additional references, Nature Portfolio reporting summaries, source data, extended data, supplementary information, acknowledgements, peer review information; details of author contributions and competing interests; and statements of data and code availability are available at 10.1038/s41591-024-02929-4.

### Supplementary information

Supplementary Information Supplementary Figs. 1–3 and STROBE checklist.

Reporting Summary

## Figures and Tables

**Extended Data Table 1:**
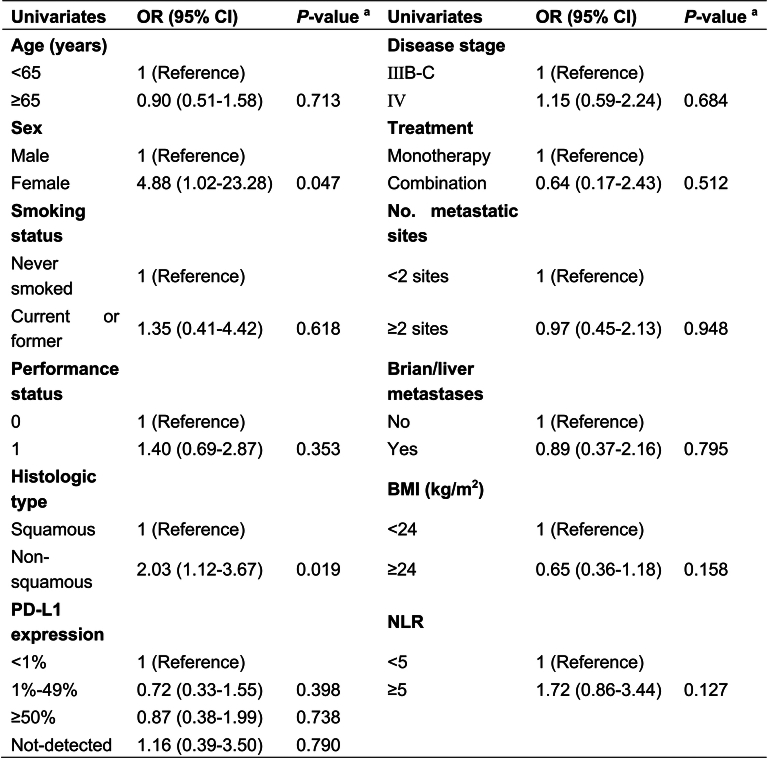
The multivariable logistic regression analysis for estimating the likelihood of emotional distress

^a^*P* values were calculated using two-sided multivariable logistic regression. Abbreviations: OR, odds ratio; PD-L1, programmed death ligand-1; BMI, body mass index; NLR, neutrophil to lymphocyte ratio; CI, confidence interval.

**Extended Data Table 2:**
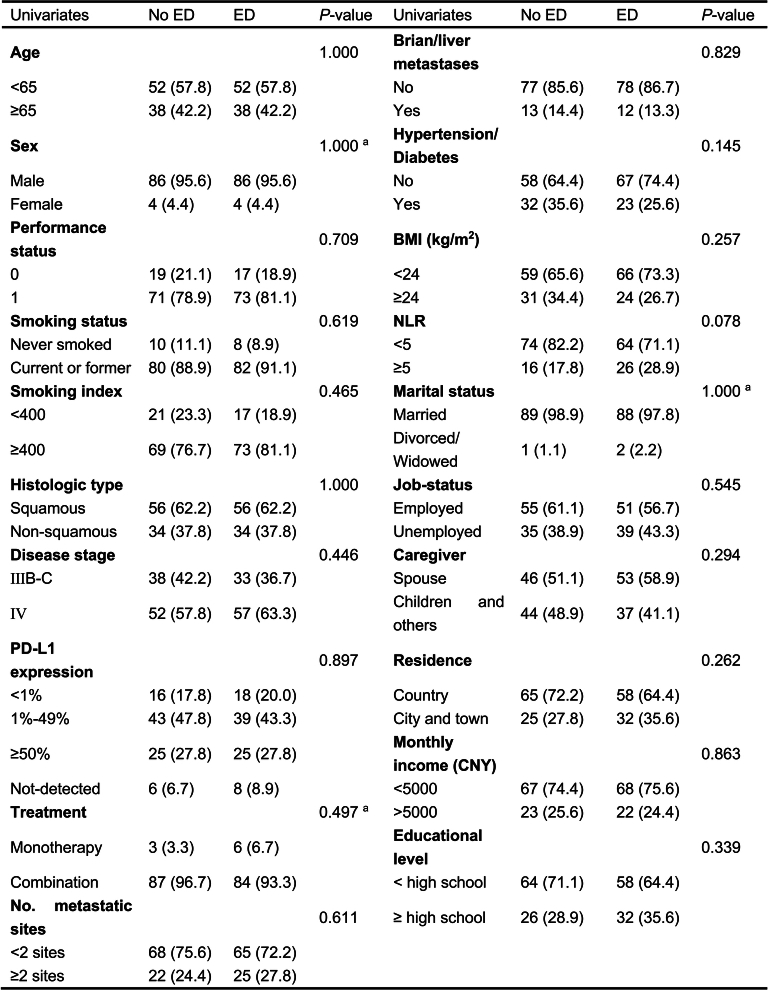
Baseline characteristics after propensity-score matching

^a^Proportion comparisons analyses (sex, marital status and treatment) were calculated using a two-sided Fisher’s exact test. Proportion comparisons of the other characteristics were calculated using a two-sided χ^2^ test. ED, emotional distress (n = 90), no ED, no emotional distress (n = 90); Abbreviations: PD-L1, programmed death ligand-1; BMI, body mass index; NLR, neutrophil to lymphocyte ratio; CNY, Chinese Yuan.

**Extended Data Table 3:**
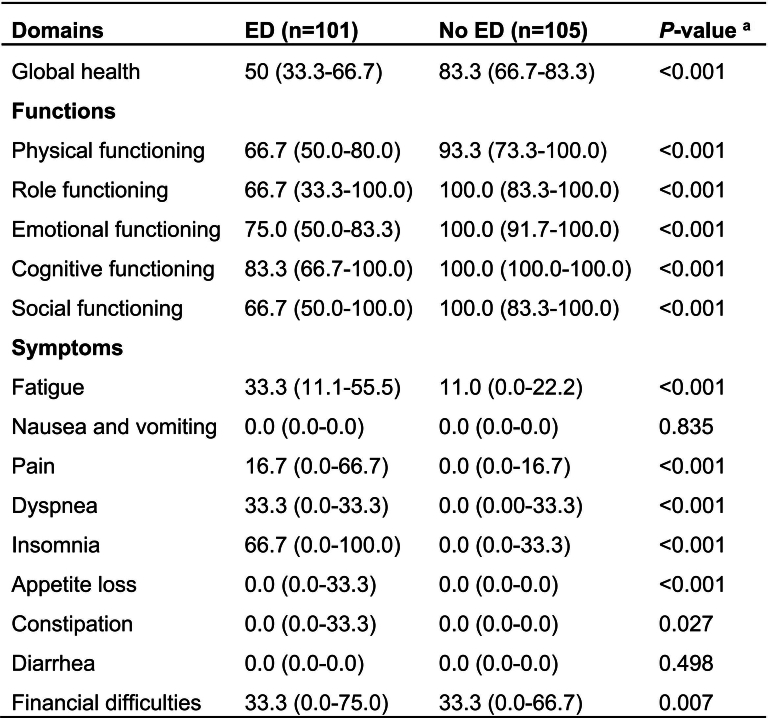
The association between emotional distress and quality of life

^a^*P* values were calculated using a two-sided Mann-Whitney U test. The data were described by the median (IQR). Abbreviation: ED, emotional distress.

## Data Availability

The datasets generated and/or analyzed during the current study are not publicly available due to patient confidentiality and proprietary considerations. To minimize the risk of patient reidentification, deidentified individual patient-level clinical data are available under restricted access. All requests for datasets should be directed to principal investigator F.W. (wufang4461@csu.edu.cn) and will be responded to within 8 weeks. Requests will be reviewed by the Second Xiangya Hospital to determine whether the request is subject to any intellectual property or confidentiality obligations, thereby deciding whether the data can be provided. Patient-related data require the requesting researcher to sign a data access agreement with the Second Xiangya Hospital, and data will be shared in aggregate form if there is not a reasonable likelihood of participant reidentification.
