# Supplementary material for: Association between pretreatment emotional distress and immune checkpoint inhibitor response in non-small-cell lung cancer
Source: Nat Med. 2024 May 13;30(6):1680–8. doi: 10.1038/s41591-024-02929-4 (PMC11186781; doi:10.1038/s41591-024-02929-4)
Supplement: Supplementary file 2 — Reporting Summary [file 41591_2024_2929_MOESM2_ESM.pdf]

nature portfolio

Corresponding author(s): Fang Wu
Last updated by author(s): Mar 17, 2024

Reporting Summary

Nature Portfolio wishes to improve the reproducibility of the work that we publish. This form provides structure for consistency and transparency in reporting. For further information on Nature Portfolio policies, see our Editorial Policies and the Editorial Policy Checklist.

Statistics

For all statistical analyses, confirm that the following items are present in the figure legend, table legend, main text, or Methods section.

n/a	Confirmed	
☐	☒	The exact sample size (*n*) for each experimental group/condition, given as a discrete number and unit of measurement
☒	☐	A statement on whether measurements were taken from distinct samples or whether the same sample was measured repeatedly
☐	☒	The statistical test(s) used AND whether they are one- or two-sided<br>*Only common tests should be described solely by name; describe more complex techniques in the Methods section.*
☐	☒	A description of all covariates tested
☐	☒	A description of any assumptions or corrections, such as tests of normality and adjustment for multiple comparisons
☐	☒	A full description of the statistical parameters including central tendency (e.g. means) or other basic estimates (e.g. regression coefficient) AND variation (e.g. standard deviation) or associated estimates of uncertainty (e.g. confidence intervals)
☐	☒	For null hypothesis testing, the test statistic (e.g. *F*, *t*, *r*) with confidence intervals, effect sizes, degrees of freedom and *P* value noted<br>*Give P values as exact values whenever suitable.*
☒	☐	For Bayesian analysis, information on the choice of priors and Markov chain Monte Carlo settings
☒	☐	For hierarchical and complex designs, identification of the appropriate level for tests and full reporting of outcomes
☒	☐	Estimates of effect sizes (e.g. Cohen's *d*, Pearson's *r*), indicating how they were calculated

Our web collection on statistics for biologists contains articles on many of the points above.

Software and code

Policy information about availability of computer code

Data collection	No software was used for data collection.
Data analysis	Statistical analyses were performed using software programs IBM SPSS v24.0, R (v. 4.2.1), and R Studio (v. 1.4.1717).

For manuscripts utilizing custom algorithms or software that are central to the research but not yet described in published literature, software must be made available to editors and reviewers. We strongly encourage code deposition in a community repository (e.g. GitHub). See the Nature Portfolio guidelines for submitting code & software for further information.

Data

Policy information about availability of data

All manuscripts must include a data availability statement. This statement should provide the following information, where applicable:

- Accession codes, unique identifiers, or web links for publicly available datasets
- A description of any restrictions on data availability
- For clinical datasets or third party data, please ensure that the statement adheres to our policy

The datasets generated and/or analyzed during the current study are not publicly available due to patient confidentiality and proprietary considerations. To minimize the risk of patient re-identification, de-identified individual patient-level clinical data is available under restricted access. All requests for datasets should be directed to principal investigator F.W (wufang4461@csu.edu.cn) and will be responded to within 8 weeks. Requests will be reviewed by the Second Xiangya Hospital to determine whether the request is subject to any intellectual property or confidentiality obligations, thereby deciding whether the data can be provided.

Patients-related data require the requesting researcher to sign a data access agreement with the Second Xiangya Hospital, and data will be shared in aggregate form if there is not a reasonable likelihood of participant reidentification.

Research involving human participants, their data, or biological material

Policy information about studies with human participants or human data. See also policy information about sex, gender (identity/presentation), and sexual orientation and race, ethnicity and racism.

Reporting on sex and gender	Data on biological sex (female or male) was collected and reported in the manuscript. The study included female (n=17) and male (n=210) patients, with sex being summarized as part of the demographic characteristics in Table 1. Sex was not considered in the study design, and both male and female patients were eligible. The emotional distress group had a higher proportion of females than the no emotional distress group. To address potential confounding effects, we utilized propensity score matching (PSM) and inverse probability of treatment weighting (IPTW) to adjust the distribution of sex. Subgroup analysis was performed to explore the association between emotional distress and progression-free survival (PFS) in female and male subgroups. This study demonstrated a high percentage of male subjects, which can be attributed to the fact that never-smoking females in Chinese often have a higher rate of EGFR/ALK/ROS1 gene positivity, and the distribution of sex in our study aligned with clinical studies in the Chinese population (Lu, S., et al. JAMA 2024). No data on gender (social attribute) were collected.
Reporting on race, ethnicity, or other socially relevant groupings	No data on race, ethnicity, or other socially relevant groupings were collected.
Population characteristics	Stage IIIB–IV NSCLC patients aged 18 years or older, with an ECOG performance status of 0-1, and at least one measurable lesion according to RECIST v1.1, were eligible to receive ICIs or combination therapy with chemotherapy as first-line treatment. A total of 227 patients with NSCLC were ultimately included for analysis. The majority of patients were male (92.5%), diagnosed with stage IV disease (58.1%), a PS score of 1 (81.1%), aged below 65 years (56.8%), smokers (87.2%), and pathology of lung squamous carcinoma (61.7%).
Recruitment	We conducted screening among inpatients newly diagnosed with stage IIIB-IV NSCLC at the Second Xiangya Hospital of Central South University. Patients were recruited by participating investigators. Patients meeting the inclusion criteria and not having any exclusion criteria were enrolled after obtaining written informed consent. No other bias emerging from recruitment is expected.
Ethics oversight	The study was approved by the Ethics Committee of the Second Xiangya Hospital of Central South University.

Note that full information on the approval of the study protocol must also be provided in the manuscript.

Field-specific reporting

Please select the one below that is the best fit for your research. If you are not sure, read the appropriate sections before making your selection.

☒ Life sciences ☐ Behavioural & social sciences ☐ Ecological, evolutionary & environmental sciences

For a reference copy of the document with all sections, see nature.com/documents/nr-reporting-summary-flat.pdf

Life sciences study design

All studies must disclose on these points even when the disclosure is negative.

Sample size	The patients with advanced NSCLC were recruited between June 1, 2021 and July 31, 2023, and ultimately 227 patients were included for analysis. As this is an observational study, no power calculation for sample size was conducted. To the best of our knowledge, this is the largest sample size investigating on the association between emotional distress and efficacy of immunotherapy in lung cancer. These sample sizes were found to be sufficient, supported by the significance of the primary endpoint.
Data exclusions	For the analyses of clinical efficacy, no patients were excluded. For the analysis of quality of life, 21 patients were excluded because they did not complete the quality of life questionnaire. In the exploratory analysis, 187 patients completed both the baseline and Time 2 assessments of emotional distress and were included in the emotional dynamic analysis. Additionally, 210 patients underwent testing for serum cortisol and ACTH at baseline, all of whom were included in the stress hormone analysis.
Replication	Replication is not applicable as this is an observational study. Here we report the data of association between emotional distress and the efficacy of first-line treatment of ICIs in advanced NSCLC (STRESS-LUNG-1).
Randomization	Randomization is not performed as this is a prospective observational cohort study with grouping based on the emotional distress states of the patients.
Blinding	Blinding was not performed as this is a prospective observational cohort study with no randomization and all enrolled patients were allocated to receive the first-line therapy of ICIs.

Reporting for specific materials, systems and methods

We require information from authors about some types of materials, experimental systems and methods used in many studies. Here, indicate whether each material, system or method listed is relevant to your study. If you are not sure if a list item applies to your research, read the appropriate section before selecting a response.

Materials & experimental systems

n/a	Involved in the study	
☒	☐	Antibodies
☒	☐	Eukaryotic cell lines
☒	☐	Palaeontology and archaeology
☒	☐	Animals and other organisms
☐	☒	Clinical data
☒	☐	Dual use research of concern
☒	☐	Plants

Methods

n/a	Involved in the study	
☒	☐	ChIP-seq
☒	☐	Flow cytometry
☒	☐	MRI-based neuroimaging

Clinical data

Policy information about clinical studies

All manuscripts should comply with the ICMJE guidelines for publication of clinical research and a completed CONSORT checklist must be included with all submissions.

Clinical trial registration

NCT05477979

Study protocol

The study design has been meticulously described on ClinicalTrials.gov. The full of 'study protocol' is not available since the Methods section of manuscript encompasses comprehensive details, including study design, participants, treatment procedures, data collection and evaluation, evaluation of emotional distress, assessment of quality of life, stress hormone determination, and statistics analysis, thus offering a lucid and detailed elucidation of the study protocol.

Data collection

Patients were recruited from June 1, 2021 to July 31, 2023 at the Second Xiangya Hospital of Central South University. The data cut-off in the article was November 30, 2023. All efficacy data were collected at the participating center.

Outcomes

The primary endpoint of the study is the investigators assessed progression-free survival (PFS). PFS was defined as the duration between the date of initiation of ICIs and disease progression or death, whichever occurred first. The secondary endpoints include the objective response rate (ORR), overall survival (OS), and quality of life (QoL). The ORR was determined by calculating the percentages of patients who exhibited a confirmed complete response, defined as the disappearance of all lesions and pathological lymph nodes with a reduction in short axis to <10 mm, and a partial response, characterized by at least a 30% decrease in the size of target lesions, based on RECIST v1.1 criteria. OS refers to the duration between the date of initiation of ICIs and death from any cause. Quality of life was assessed using the EORTC QLQ-C30. Exploratory outcomes involve the analysis of emotional distress dynamics and the relationships between peripheral blood distress biomarkers and the efficacy of ICI treatments.

Plants

Seed stocks

Not applicable.

Novel plant genotypes

Not applicable.

Authentication

Not applicable.
